# Role of Vesicle-Associated Membrane Protein-Associated Proteins (VAP) A and VAPB in Nuclear Egress of the Alphaherpesvirus Pseudorabies Virus

**DOI:** 10.3390/v13061117

**Published:** 2021-06-10

**Authors:** Anna D. Dorsch, Julia E. Hölper, Kati Franzke, Luca M. Zaeck, Thomas C. Mettenleiter, Barbara G. Klupp

**Affiliations:** 1 Institute of Molecular Virology and Cell Biology, Friedrich-Loeffler-Institut, 17493 Greifswald, Insel Riems, Germany; anna_dorsch_187@web.de (A.D.D.); julia.hoelper@fli.de (J.E.H.); luca.zaeck@fli.de (L.M.Z.); thomasc.mettenleiter@fli.de (T.C.M.); 2 Institute of Infectology, Friedrich-Loeffler-Institut, 17493 Greifswald, Insel Riems, Germany; kati.franzke@fli.de

**Keywords:** herpesvirus, pseudorabies virus, PrV, nuclear egress, vesicle-associated membrane protein associated protein, VAPA, VAPB, CRISPR/Cas9 genome editing

## Abstract

The molecular mechanism affecting translocation of newly synthesized herpesvirus nucleocapsids from the nucleus into the cytoplasm is still not fully understood. The viral nuclear egress complex (NEC) mediates budding at and scission from the inner nuclear membrane, but the NEC is not sufficient for efficient fusion of the primary virion envelope with the outer nuclear membrane. Since no other viral protein was found to be essential for this process, it was suggested that a cellular machinery is recruited by viral proteins. However, knowledge on fusion mechanisms involving the nuclear membranes is rare. Recently, vesicle-associated membrane protein-associated protein B (VAPB) was shown to play a role in nuclear egress of herpes simplex virus 1 (HSV-1). To test this for the related alphaherpesvirus pseudorabies virus (PrV), we mutated genes encoding VAPB and VAPA by CRISPR/Cas9-based genome editing in our standard rabbit kidney cells (RK13), either individually or in combination. Single as well as double knockout cells were tested for virus propagation and for defects in nuclear egress. However, no deficiency in virus replication nor any effect on nuclear egress was obvious suggesting that VAPB and VAPA do not play a significant role in this process during PrV infection in RK13 cells.

## 1. Introduction

Besides being important pathogens, viruses are also renowned as pioneers in cell biology. Numerous basic principles in molecular cell biology have been identified using viruses or viral proteins [1]. Among these pathways is the vesicular transport of (viral) glycoproteins from the endoplasmic reticulum (ER) through the Golgi towards the plasma membrane [2]. This vesicle-mediated transport pathway, which encompasses a series of membranous compartments in the eukaryotic cell, has been intensively studied and is already understood in detail. Surprisingly, the nuclear envelope seems to be excluded from this rather ubiquitous vesicular transport, and it was long believed that all exchange of material between the nucleus and the cytoplasm occurs exclusively through the nuclear pores [3].

Herpesviruses are complex viruses and their double-stranded DNA genome encodes for at least 70 different viral proteins, including enzymes involved in nucleotide metabolism and DNA replication [4]. For virus replication and assembly, herpesviruses not only use the cytoplasm but also the host cell nucleus. In the nucleus, viral transcription and DNA replication take place. The newly synthesized viral DNA is incorporated in the nucleus into a preformed capsid, which is assembled autocatalytically from capsid subcomplexes. The mature nucleocapsid, however, is far too bulky to pass through the nuclear pores. Therefore, herpesviruses are compelled to use other means to overcome the nuclear envelope barrier to access the site of final virion maturation [5]. Although expansion of nuclear pores has been suggested to occur during herpesvirus infection [6,7], the common pathway involves a vesicle-mediated process, which is designated as envelopment–de-envelopment pathway [8,9]. Nucleocapsids bud at the inner nuclear membrane (INM), thereby acquiring an envelope, which results in a primary virion located in the perinuclear space (PNS). Subsequently, in a still largely enigmatic process, the primary envelope fuses with the outer nuclear membrane (ONM) to finally release the nucleocapsid into the cytoplasm (reviewed in [10,11,12]). Once in the cytoplasm, tegument proteins are added and the final virion envelope is acquired from membranes of trans-Golgi network-derived vesicles or endocytic membranes [8,13]. Our research on the nuclear egress of pseudorabies virus (PrV) in rabbit kidney cells (RK13) was one of the first to describe this process on a molecular basis and added important biochemical and structural data supporting the envelopment–de-envelopment pathway, which was long disputed for herpes simplex virus 1 (HSV-1) (reviewed in [8]).

The nuclear egress complex (NEC) mediates the first step in this vesicle-mediated translocation (reviewed in [10,11,12]). Two conserved viral proteins designated as pUL34 and pUL31 in the alphaherpesviruses HSV-1 and PrV form the NEC. pUL34 is a type II, tail-anchored membrane protein, which is autonomously targeted to the nuclear envelope whereas pUL31 is channeled into the nucleus through nuclear pores using the cellular import machinery [14,15,16,17]. Both proteins interact at the nucleoplasmic site of the INM to form the dimeric NEC. Oligomerization of NEC dimers into hexons and further into honeycomb-like lattices is thought to induce membrane bending and finally scission of vesicles into the PNS (reviewed in [18]). This process can be induced in eukaryotic cells by co-expressing only the two NEC components [19,20], indicating that no other viral protein is necessary. Vesicle formation from synthetic membranes can also be mediated by co-expression of only pUL31 and pUL34 demonstrating that also no other cellular protein is required for this vesiculation process [21,22]. However, fusion of these vesicles is only very rarely observed, indicating that other proteins are involved in an efficient de-envelopment.

A viral protein already known to participate in this fusion process is the alphaherpesvirus specific protein kinase pUS3. In the absence of pUS3 or its kinase function, primary enveloped virions accumulate in the PNS within large herniations of the INM [23,24,25,26,27]. Enzymatically active pUS3, however, is not essential for alphaherpesvirus replication and progeny virus titers are only slightly reduced in its absence, indicating that phosphorylation of viral and/or cellular proteins may modulate efficiency of nuclear egress. Since no other viral proteins were identified to play a major role, we speculated that a cellular fusion machinery acting at the nuclear envelope is hijacked by herpesviruses [10]. Unfortunately, knowledge on fusion proteins or fusion machineries active at or within the nuclear membranes is still limited.

SUN2, a component of the linker of the nucleoskeleton and cytoskeleton (LINC) complex regulating the spacing between the nuclear membranes [28], was shown to modulate fusion of the primary virion envelope with the ONM. Overexpression of a dominant-negative SUN2 resulted in accumulations of primary virions in a dilated PNS, indicating that SUN2 in the LINC complex keeps the membranes at a certain distance to allow for efficient membrane fusion [29]. In line with this, an accumulation of primary virions was found in cells deficient for the AAA + ATPases Torsin A and B with a possible impact on scission of the primary enveloped particles from the INM [30]. Torsin A is reported to be involved in LINC complex assembly and/or disassembly [31,32], again pointing to a role for the LINC complex in nuclear egress.

Knockdown of CD98 heavy chain and its binding partner ß1 integrin resulted in accumulations of primary HSV-1 virions similar to those found in the absence of pUS3 [33] or in simultaneous absence of glycoproteins (g)B and gH [34], which are part of the core fusion machinery required for virus entry (reviewed in [35]). CD98 heavy chain was shown to interact with gB, gH, pUL31 and pUS3 and it was proposed that this interaction drives membrane fusion between the primary envelope and the ONM [33]. For PrV, however, gB and gH play no role in nuclear egress and virion morphogenesis occurs independently of these glycoproteins. In addition, neither of these glycoproteins could be detected in primary virions or in the INM further arguing against a functional role in de-envelopment [36]. This evidence renders a similar function for CD98 in nuclear egress of PrV nucleocapsids unlikely, but this remains to be tested experimentally.

The endosomal sorting complex required for transport (ESCRT) III has also been described to be involved in nuclear egress of HSV-1. In infected cells, ESCRT-III is recruited to the INM and scission of primary enveloped particles is impaired in ESCRT-III depleted cells [37,38], whereas another study showed no defect in nuclear egress by using dominant negative versions of several ESCRT components [39]. However, none of the above-mentioned cellular proteins is essential for nuclear egress of herpesvirus nucleocapsids.

In a recent study, the cellular vesicle fusion protein vesicle-associated membrane protein (VAMP)-associated protein B (VAPB) was shown to play a role in nuclear egress of HSV-1 [40]. Mass spectrometric analysis of membranous vesicles isolated from infected HeLa cells identified several cellular proteins known to be involved in vesicular trafficking in the cytoplasm. Of those, VAPB, which showed the highest enrichment score, was further analyzed [40]. Using immune labeling, VAPB was found to co-localize with the NEC component pUL34 in the nuclear membrane and knockdown of VAPB by siRNA resulted in an around two-log_10_ drop in virus titers accompanied by an accumulation of viral particles in the PNS, strongly suggesting a role in nuclear egress [40]. Moreover, expression of a mutant VAPB, which is found in patients suffering from amyotrophic lateral sclerosis, resulted in a dilation of the PNS with membrane vesicles accumulating in the PNS pointing to a function in nuclear envelope maintenance [41].

VAPB and VAPA are members of the highly conserved VAP family and are ubiquitously expressed in eukaryotic cells of various tissues and organs (reviewed in [42]). The less well characterized VAPC is a differently spliced isoform of VAPB sharing 70 N-terminal amino acids followed by additional 29 unique amino acids [42]. VAPs play a role in diverse cellular functions, such as regulation of lipid transport and homeostasis, and membrane trafficking. VAPs are mainly localized in the ER, where they interact with many different proteins to tether the ER to other intracellular organelles at defined membrane contact sites. A possible role of VAPs in membrane fusion is based on their interaction with SNARE (soluble N-ethylmaleimide-sensitive factor attachment protein receptor) proteins, but the structural and functional role of this interaction remains to be established (reviewed in [42]). VAPA and VAPB are type II membrane proteins with their C-terminal end anchored in the membrane. Thus, a major part of the protein extends into the cytosol when anchored in the ER or into the nucleoplasm when they reach the INM [40]. In mammalian cells, homo- and heterodimeric complexes of VAPA and VAPB have been described [43,44] pointing to an at least partial redundancy.

In addition to HSV-1, members of the VAP-family have already been described to play an important role for replication of different viruses. For example, VAPA interacts with the nonstructural protein 1 (NS1) of human norovirus and replication efficiency of murine norovirus was significantly decreased in VAPA or VAPB deficient cells [45]. It was also shown that NS5A and NS5B of hepatitis C virus interact with VAPB and that this interaction is essential for virus replication most likely by providing a scaffold in the appropriate membrane compartment [42,46].

Here, we analyzed the role of VAPB and VAPA for replication of PrV. Although HSV-1 and PrV belong to the alphaherpesvirus subfamily, they differ in several aspects of the viral life cycle. Regarding nuclear egress, HSV-1 seems to add a number of tegument proteins already in the nucleus, e.g., pUL47 and pUL51. These proteins could not be detected on nuclear capsids of PrV (reviewed in [47]) [48], indicating that in addition to the conserved alphaherpesviral nuclear egress proteins pUL34, pUL31 and pUS3, these proteins might recruit a different set of cellular proteins to modulate nuclear egress.

To test for the involvement of VAPs in nuclear egress of PrV, we targeted the corresponding genes by CRISPR/Cas9-based genome editing in RK13 cells. RK13 cells have been our standard PrV host cells for more than 20 years, revealing basic principles of herpesvirus morphogenesis and release (reviewed in, e.g., [8,10,49]). RK13 cell clones with deletions in *VAPB*, *VAPA* or both coding genes were tested for efficient replication of PrV and for impairment of nuclear egress. However, in contrast to HSV-1 in HeLa cells, neither single knockout (KO) of VAPB, VAPA nor VAPA/B double KO (DKO) had a significant effect on PrV replication or on nuclear egress in RK13 cells.

## 2. Materials and Methods

### 2.1. Cells and Virus

RK13 (CCLV-Rie 0109), HeLa (CCLV-Rie 0082) and Vero (CCLV-Rie 0228) cells were grown in minimum essential medium supplemented with 10 % fetal calf serum at 37 °C and 5 % CO_2_ in a humid atmosphere. PrV strain Kaplan (PrV-Ka; [50]) was propagated in RK13 cells.

### 2.2. CRISPR/Cas9-Based Gene Editing

The transcript ID for the corresponding genes in the host species *Oryctolagus cuniculus* (OryCun2.0) was identified using the genome browser database e!Ensembl (www.ensembl.org) [51]. Entering the transcript ID into the online program ChopChop (chopchop.cbu.uib.no [52]), specific gRNAs targeting exon 2 in the corresponding genes were designed. To generate knockout cell lines, single guide RNA (sgRNAs) expressing plasmids were constructed. Three or four sgRNAs (each 20 nucleotides) were selected per gene and oligonucleotides were ordered in forward and reverse orientation (Eurofins, Ebersberg, Germany) with overhangs compatible with the BbsI restriction enzyme site. In addition, primers to amplify the target gene regions were synthesized (Table 1).

The corresponding forward and reverse oligonucleotides were hybridized, phosphorylated and cloned into BbsI-cleaved vector pX330-NeoR [53]. Correct synthesis and cloning were verified by sequencing using primer HU6-F (Table 1). All three or four sgRNA expressing plasmids were co-transfected into RK13 cells by calcium phosphate co-precipitation [54] using equal amounts of each plasmid. For VAPA/B DKO cells, all seven plasmids were transfected simultaneously.

From each transfection assay, eleven G418-resistant single cell colonies were selected and screened for an effect on virus propagation. For this, cells were seeded in 24 well dishes and infected with PrV-Ka at a multiplicity of infection (MOI) of 5. Cells were harvested after 24 h and progeny titers were determined on RK13 cells. Since titers derived from the different single cell clones showed no clear difference, two or three clones each were randomly selected. The targeted gene region was amplified, and the PCR product was sequenced. For this, genomic DNA was isolated by lysing the cells in TEN/Sarkosyl buffer (20 mM TrisHCl pH 7.4, 1 mM EDTA, 150 mM NaCl, 3% N-lauroylsarcosinate) at 65 °C for 20 min. Then, RNA and proteins were digested by treatment with RNase (Roche Diagnostics, Mannheim, Germany) for 30 min at 37 °C followed by incubation with Pronase (Serva, Heidelberg, Germany) for 2 h at 45 °C. DNA was extracted by phenol/chloroform, precipitated with ethanol, and resuspended in Tris-HCl, pH 7.4. Gene specific primers (Table 1) were used to amplify the target gene region using either Phusion^®^ High Fidelity DNA Polymerase (ThermoFisherScientific, Darmstadt, Germany) or the KAPA HiFi HotStart Ready Mix PCR Kit (Roche Diagnostics) as recommended by the manufacturers. PCR products were first sequenced directly using the corresponding forward or reverse primers and the BigDye Terminator Cycle Sequencing Kit (ThermoFisherScientific). PCR products, whose sequence differed from the parental RK13 sequence, were cloned into the vector pBluescript SK + (Stratagene, Frankfurt am Main, Germany). As a standard, for each selected cell clone, plasmid inserts of 10 randomly picked bacterial colonies were sequenced using the vector specific T7 primer. Sequences were analyzed with Geneious Prime software (version 2019.2.3). For the in-depth analyses, one clone each, which preferably carried an out-of-frame mutation in the targeted exon on both alleles was used.

### 2.3. Western Blot Analysis

Lysates of parental, single and double knockout RK13 as well as of Vero and HeLa cells were separated in sodium dodecyl sulfate (SDS)-10% polyacrylamide gels. Proteins were transferred to nitrocellulose membranes and blocked with 6% skimmed milk. Parallel blots were incubated with the polyclonal rabbit sera specific for VAPA (1:2.500; Invitrogen # PA5-22188; raised against a synthetic peptide corresponding to a region within amino acids 29 and 122 of human VAPA ID#Q9P0L0; ThermoFisherScientific), VAPB (1:5.000; Invitrogen # PA5-53023; corresponding to amino acids 105-216 of human VAPB ID#O95292; ThermoFisherScientific), or with a monoclonal α-tubulin antibody (1:10.000; Sigma, Taufkirchen, Germany). Bound antibodies were detected after incubation with peroxidase-conjugated anti-rabbit or anti-mouse antibodies (Dianova, Hamburg, Germany) using the Clarity Western ECL substrate (Bio-Rad Laboratories, Feldkirchen, Germany). Signals were recorded with a VersaDoc 4000 MP imager (Bio-Rad Laboratories) using the Quantity One software (version 4.6.9).

### 2.4. Actin Staining

To visualize a putative reorganization of actin in cells lacking VAPA/B as reported previously for HeLa cells [55], RK13 as well as the single and double KO cells were fixed with 4% paraformaldehyde for 20 min followed by permeabilization with 0.1% Triton-X-100. Actin was labelled with phalloidin-Alexa Fluor 488 (dilution 1:50; ThermoFisherScientific) and nuclei were counterstained with DAPI. Samples were imaged with a confocal laser scanning microscope (Leica DMI 6000 TCS SP5, 63× oil-immersion objective, NA = 1.4; Leica, Wetzlar, Germany). Image processing and visualization were done with Fiji (v1.53) [56].

### 2.5. Growth Properties

Cell clones carrying mutations in the targeted genes were tested for efficient PrV propagation. To this end RK13-VAPA KO, RK13-VAPB KO, RK13-VAPA/B DKO and parental RK13 cells were infected with PrV-Ka using either a high MOI of 5 or a low MOI of 0.05. For this, precooled cells and diluted virus suspensions were incubated on ice for 1 h followed by addition of prewarmed medium to allow for synchronous infection. Non-penetrated virus was inactivated by low pH treatment after 1 h at 37 °C [57]. Cells and supernatant were harvested 24 h and 48 h post infection and titers were determined on RK13 cells. Mean values of five independent assays were calculated and plotted with the corresponding standard deviation. No statistically significant difference in virus titers was found using a two-way ANOVA with Dunnett’s multiple comparison test performed with GraphPad Prism (version 9.0.0.121).

### 2.6. Ultrastructural Analysis

RK13, RK13-VAPA KO, RK13-VAPB KO and RK13-VAPA/B DKO cells were infected with PrV-Ka at an MOI of 1 and processed for electron microscopy 14 h post infection as described recently [29].

## 3. Results

### 3.1. Generation of RK13-VAPB KO, RK13-VAPA KO and RK13-VAPA/B DKO Cell Lines

CRISPR/Cas9-based genome editing is a highly efficient tool to target any gene within a given host genome for which sequence information is available. We used the online tool ChopChop (chopchop.cbu.uib.no [52]) to select either four or three sgRNAs targeting the second exon of VAPA or VAPB with the lowest probability for off-site targets. Oligonucleotides were cloned into the plasmid pX330-NeoR, which encodes the Cas9 nuclease and a resistance for neomycin/G418 [53]. RK13 cells were co-transfected with the four or three sgRNA-expressing plasmids or with all seven plasmids simultaneously to generate single and double knockout cells, respectively. Transfected cells were selected in medium containing 0.5 mg/mL G418 and resistant single cell colonies were analyzed. Based on the data shown for HSV-1 [40], we expected that at least RK13-VAPB KO would be easily identifiable by a drop in progeny virus titers compared to parental RK13 cells. For each transfection assay, 11 cell clones were first screened for efficient virus propagation. Since differences in progeny virus titers were only marginal (data not shown), two or three randomly selected cell clones from the originally tested 11 single cell clones were further analyzed. For this, the CRISPR/Cas9-targeted region was amplified by PCR with genomic DNA as template and sequenced. One cell clone each preferentially with an out-of-frame mutation and/or a large deletion in the coding region of the targeted gene were further tested.

**Figure 1:**
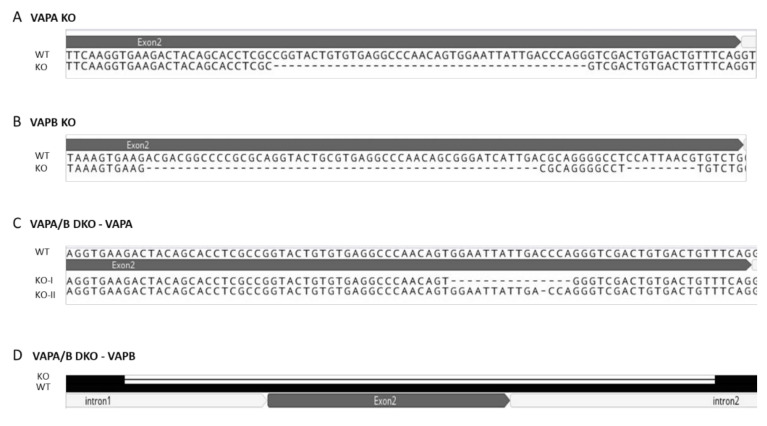
CRISPR/Cas9 genome editing of VAPA- and VAPB-encoding genes in RK13 cells. Shown are the wildtype sequences (WT) and the deletions uncovered in the respective genes of the selected knockout cells (KO). In RK13-VAPA KO cells, 41 nt were deleted in the *VAPA* ORF (**A**), while 50 nt and additional 9 nt were removed in the VAPB coding region in RK13-VAPB KO (**B**). In both single KO cell lines, the alleles comprise identical changes. *VAPA* in RK13-VAPA/B double knockout (DKO) contained deletions of 15 nt (KO-I) or 1 nt (KO-II) (**C**), while the complete exon 2 in *VAPB* is missing (**D**). Due to the large deletion identical regions are indicated by black bars and the deleted sequence is represented by a thin line.

As shown in Figure 1, the selected clone RK13-VAPA KO carried a deletion of 41 nucleotides (nt) in exon 2 of *VAPA*, while in exon 2 of RK13-VAPB KO a 50 nt and an additional 9 nt stretch were deleted. In the cloned PCR products from genomic DNA of both cell lines, only one type of mutation was found indicating that the mutation is biallelic. Sequencing of the RK13-VAPA/B DKO cell line revealed a huge deletion comprising 379 nt in *VAPB* thereby removing the complete exon 2 including parts of the intron regions, but only a short deletion of either 15 nt or only one nucleotide in exon 2 of *VAPA* (Figure 1).

### 3.2. Characterization of RK13-VAPB KO, RK13-VAPA KO and RK13-VAPA/B DKO

**Figure 2:**
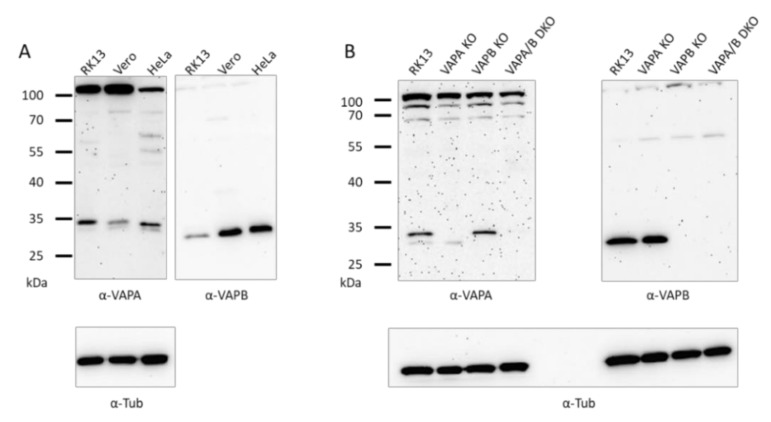
Immunoblots showing VAPA and VAPB in RK13, Vero and HeLa cells and the absence of VAPA and/or VAPB in the corresponding RK13 KO and DKO cell lines. Proteins in lysates of RK13, Vero and HeLa cells (**A**) or RK13 and the single as well as the double VAP KO cell lines (**B**) were separated on SDS-10 % polyacrylamide gels, and parallel blots were incubated with polyclonal sera against VAPA or VAPB. Masses of marker proteins are given on the left in kDa. As loading control, parallel (**A**) or the same blots were (re-)probed with anti-α-tubulin.

The selected single and double KO cell lines all carried deletions in the targeted gene region. To test for protein expression, immunoblotting was performed. Reactivity of the VAPA- and VAPB-specific antisera with the rabbit homologs in RK13 cells was compared to Vero and HeLa cells. As presented in Figure 2A, proteins of comparable electrophoretic mobility as in Vero and HeLa cells could be detected in RK13 cells, highlighting the high conservation of VAPs (reviewed in [42]). It is difficult to speculate on the relative expression levels since the sera might detect the homologs with different affinities. However, the comparable α-tubulin-specific signal indicates that a similar amount of cell lysate was applied.

To test whether the corresponding proteins are missing in the knockout cells, lysates of RK13, RK13-VAPA KO, RK13-VAPB KO and RK13-VAPA/B DKO cells were separated on SDS-10 %-polyacrylamide gels and transferred to nitrocellulose. Parallel blots were probed with polyclonal rabbit antisera specific for VAPA or VAPB. A signal corresponding to VAPA, as detected in Vero, HeLa and the parental RK13 cells (Figure 2A), is missing in RK13-VAPA KO and RK13-VAPA/B DKO cells. The 33 kDa VAPB band is absent in lysates of RK13-VAPB KO and RK13-VAPA/B DKO cells (Figure 2B), indicating that the gene specific knockout was successful in eliminating protein expression in the selected cell clones. The faint signal visible in immunoblots with the VAPA-specific serum might be due to a weak cross-reactivity with VAPB, while the upper bands represent unspecific signals since they are identical in all cell lines. As loading control, both blots were re-probed with anti-α-tubulin.

**Figure 3:**
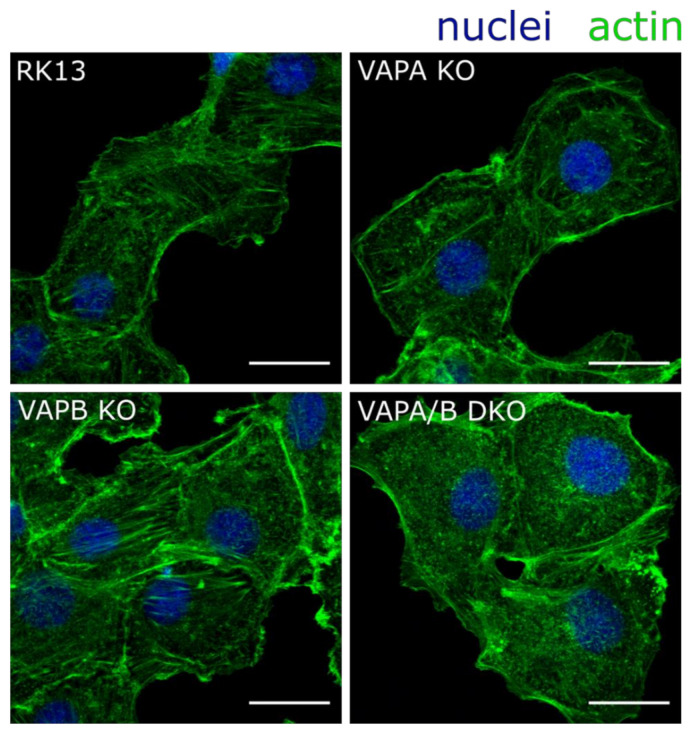
Actin organization is perturbed in RK13-VAPA/B DKO cells. RK13, RK13-VAPA KO, RK13-VAPB KO and RK13-VAPA/B DKO cells were fixed with 4% paraformaldehyde and cytoskeletal actin was stained with fluorophore-conjugated phalloidin (green). A loss of actin stress fibers and an accumulation of actin comets is obvious in RK13-VAPA/B DKO as reported for HeLa cells lacking VAPA/B [55]. Nuclei were counterstained with DAPI (blue). Shown are representative images. Scale bar = 20 µm.

Previously it was shown that actin organization is drastically perturbed in HeLa VAPA/B DKO cells [55]. To investigate whether VAPs function in a similar way in RK13 cells, we analyzed the actin skeleton organization using confocal laser-scanning microscopy. For this, we fixed and stained the parental RK13 as well as the single and double KO cells with fluorophore-conjugated phalloidin. As evident in Figure 3, stress fibers as present in RK13 and the single KO cells, are less dominant in the RK13-VAPA/B DKO cells and comparable to data shown for HeLa cells [55], supporting a functional double knockout of VAPA and VAPB. The single KO cells showed no obvious perturbation of actin, which is in line with the notion that both proteins feature at least some functional redundancy [42].

### 3.3. Absence of VAPA, VAPB or Both Has No Major Impact on PrV Replication

**Figure 4:**
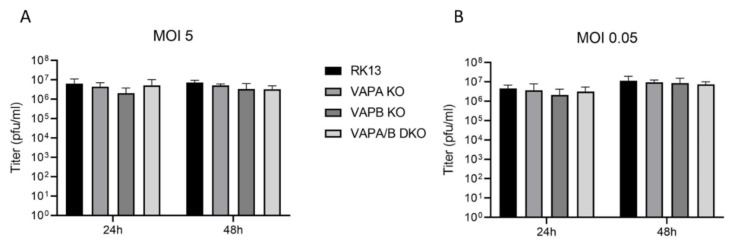
Growth properties of PrV-Ka derived from parental RK13, VAP single and double KO cells. RK13, RK13-VAPA KO, RK13-VAPB KO and RK13-VAPA/B DKO were infected with PrV-Ka at an MOI of 5 (**A**) or 0.05 (**B**) and harvested after 24 or 48 h. Shown are mean values of progeny virus titers in plaque forming units per milliliter (pfu/ml) of five replicates with the corresponding standard deviation.

To analyze the influence of the introduced modifications on replication of PrV-Ka, cells were infected either with an MOI of 5 or 0.05. Cells and supernatant were harvested 24 h or 48 h post infection and progeny viral titers were determined on RK13 cells (Figure 4). Only titers of PrV-Ka derived from RK13-VAPB KO cells were marginally lower at 24 h post infection, which, however, was not statistically significant. In addition, parallel infection of cells expressing neither VAPA nor VAPB showed no titer reduction, indicating that the minor decrease is most likely not due to the VAPB deficiency.

### 3.4. Absence of VAPA or VAPB or Both Has No Significant Impact on PrV Nuclear Egress or Final Virion Maturation

**Figure 5:**
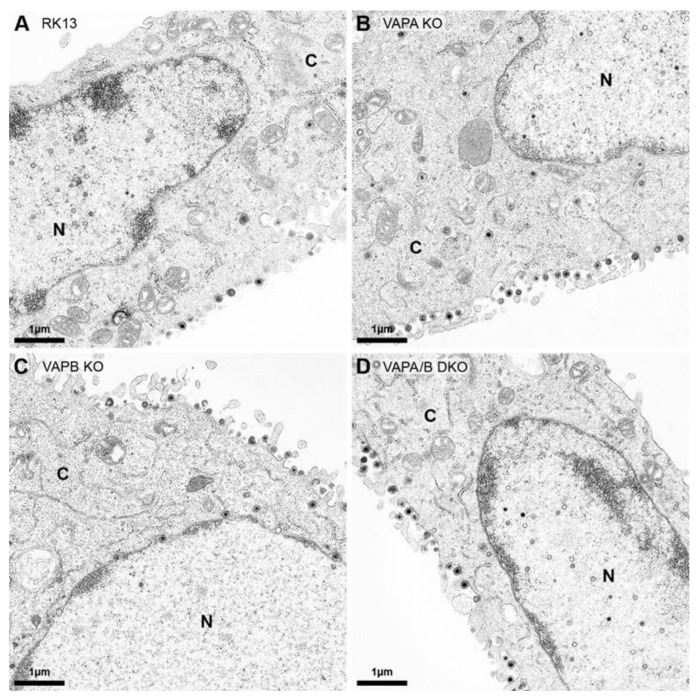
Ultrastructural analysis of PrV-Ka infected RK13, VAP KO and DKO cells. (**A**) RK13, (**B**) RK13-VAPA KO, (**C**) RK13-VAPB KO and (**D**) RK13-VAPA/B DKO cells were infected with PrV-Ka (MOI 1) and processed for electron microscopy 14 h post infection. Shown are presentative images. N: nucleus, C: cytoplasm.

The growth analyses indicated only a minor effect on titers for PrV-Ka propagated on RK13-VAPB KO cells. To investigate this in more detail and with special focus on nuclear egress, parental RK13, RK13-VAPA KO, RK13-VAPB KO and RK13-VAPA/B DKO were infected with PrV-Ka at an MOI of 1 and processed for ultrastructural analysis 14 h post infection (Figure 5). In the representative images, all stages of virion morphogenesis were observed in cells with defects in VAPA (Figure 5B), VAPB (Figure 5C) or VAPA and VAPB expression (Figure 5D), comparable to the parental RK13 cells (Figure 5A). Mature capsids in the nucleus, single primary enveloped virions in the PNS, enveloped virus particles in cytoplasmic transport vesicles and mature virions on the cell surface are easily discernible. In contrast to the study for HSV-1 after siRNA-based knockdown of VAPB in HeLa cells [40], no accumulations of capsids in the nucleus or primary virions in the PNS were obvious, indicating that neither VAPA nor VAPB individually or in combination play an important role in nuclear egress or virion morphogenesis of PrV in RK13 cells.

## 4. Discussion

The molecular mechanism of nuclear egress of herpesvirus nucleocapsids is still not fully understood. The first step in this process, the formation of primary enveloped virions by budding through the INM into the PNS, is well studied. Using a multimodal imaging approach, the NEC coat could be visualized in situ [58] and the elucidation of the crystal structures for the NEC heterodimers from several herpesviruses provided detailed insights into this characteristic vesicle formation and scission machinery at and from the INM [59,60,61,62]. In contrast, the second step encompassing fusion of the primary virion envelope with the ONM or, after escape into the lumen of the ER, with the ER membrane remains elusive. The NEC itself seems unable to mediate efficient fusion and vesicles accumulate in the PNS when both NEC components are co-expressed [19,20,21,22]. Recently, it was proposed that vesicle fusion proteins redirected from the ER to the nuclear envelope might facilitate herpesvirus nuclear egress [40]. Cell fractionation coupled with mass spectrometry found especially VAPB enriched in nuclear membrane fractions of HSV-1 infected cells. Additionally, siRNA-mediated knockdown of VAPB expression in HeLa cells resulted in approximately two-log_10_ reduced viral titers concomitant with an accumulation of virus particles in the nucleus and in the perinuclear cleft, supporting a functional role of VAPB in nuclear egress of HSV-1 [40].

These data sparked our interest to investigate the role of VAPB and VAPA in nuclear egress of PrV. VAPA and VAPB, which are highly conserved in eukaryotes, share 63 % amino acid sequence identity and are supposed to serve similar cellular functions forming not only homo- but also heterodimeric complexes [42]. We used CRISPR/Cas9 gene editing technology as provided with the vector pX330-NeoR [53] to impair VAPA and VAPB expression. We targeted the second exon of each gene by three or four different sgRNAs. The sgRNA expressing plasmids were co-transfected into RK13 cells to enhance the efficiency of the knockout and to increase the chance to introduce larger deletions, specifically aiming for out-of-frame mutations to ensure absence of protein expression. Using this approach we already generated Torsin A and Torsin B single as well as double knockout cells [30].

Preliminary testing of several single cell clones showed no drastic impact on progeny viral titers, already indicating a difference to the effect on HSV-1 after siRNA-mediated VAPB knockdown in HeLa cells [40]. Sequencing of the targeted gene region in the selected CRISPR/Cas9-mutated KO cell clones showed large biallelic out-of-frame deletions in the *VAPA* gene (41 nt) in RK13-VAPA KO and in the gene encoding VAPB (50 and 9 nt) in RK13-VAPB KO cells. In the RK13-VAPA/B DKO cells, the complete exon 2 of the *VAPB* gene, including part of the surrounding intron sequences was removed, while the *VAPA* gene contained only a single nucleotide deletion in one allele and an in-frame deletion of 15 nt in the other allele. The 5-codon in-frame deletion might still result in a truncated but functional VAPA, but immunoblot analysis using the VAPA specific serum showed no residual protein expression.

The VAPA- and VAPB-specific antisera were raised against the human proteins, but homologs are well conserved between species [42]. The VAPA-specific antiserum was generated against amino acids 29–122 of human VAPA (#Q9P0L0), which are identical in the predicted rabbit protein sequence (data not shown). The sequence of the immunogen used for generation of anti-VAPB corresponds to amino acids 105–217 of the rabbit homologue (accession no. XP_008250457) and shares 83% identical residues. Both sera specifically detected 34 and 33 kDa proteins in the parental RK13 lysates comparable to the reactivity in Vero and HeLa cells, but corresponding signals were absent in the single and double KO cells, pointing to a successful protein knockout. Loss of actin stress fibers was reported for a HeLa VAPA/B DKO cell line [55] and a comparable perturbation was found in RK13-VAPA/B DKO further supporting a successful functional knockout. The single VAP KO cells had no obvious difference in F-actin staining, indicating that loss of function can be compensated by the remaining protein.

In contrast to HSV-1 [40], growth analyses with wildtype strain PrV-Ka showed no obvious impairment in RK13 cells deficient for VAPB expression. Progeny virus titers derived from the single and VAPA/B double KO cells were comparable to parental RK13 cells independent of the MOI used and the time point analyzed. Only titers derived from RK13-VAPB KO at 24 h after infection showed a marginal, approximately, 3-fold decrease in virus progeny. Infection of RK13-VAPA/B DKO cells, which carry a large deletion in the *VAPB* gene region resulting in a complete removal of exon 2, did not show a corresponding titer reduction, arguing against a specific effect of VAPB on infectious virus production. In-depth structural analysis of infected single and double KO cells supported these results. No impairment of nuclear egress as described for HSV-1 in HeLa cells [40] was observed. In contrast, all stages of virion maturation including numerous released virions on the cell surface could be detected in agreement with the results of the single and multistep growth assays. In our preliminary studies, we tested eleven single cell clones of each of the single and double KO for an effect on progeny viral titers in parallel. However, titers were comparable for all randomly picked clones arguing against selection for second-site mutations, which may compensate a potential VAPA/B deficit.

Despite the close phylogenetic relationship between HSV-1 and PrV, previous research uncovered multiple differences between these two alphaherpesviruses. Although it is reasonable to speculate that the molecular machinery executing de-envelopment at the ONM is as conserved as is the budding and scission apparatus at the INM, various viral and host proteins seem to modulate both stages (reviewed in [47]). In growth analyses of HSV-1 on the single and double KO RK13 cells, we were unable to observe the reported two-log_10_ reduction at 48 h post infection after siRNA-mediated knockdown of VAPB in HeLa cells [40]. No titer reduction was evident for HSV-1 derived from RK13-VAPA/B DKO cells (data not shown). The reason for the different results between HSV-1 and PrV, which were generated in different cell lines and by different technical approaches, siRNA-mediated knock-down versus CRISPR/Cas9 knockout, and for HSV-1 in HeLa cells compared to RK13 cells remains to be tested. However, while VAPs might play a role in nuclear egress, they seem not to be essentially involved in the process, indicating that the key players have not been targeted yet.

Despite the prominent role of VAPs in organelle tethering, lipid homeostasis and transport, VAP-deficient cells are viable [55,63]. It has been reported that the inter-organelle membrane contact sites, in which VAPA and VAPB play a prominent role, often rely on several independent tethers to secure the essential functions in the cell [64]. In line with this, in addition to VAPA and VAPB, a third ER receptor, MOSPD2, has been identified recently by a proteomic approach [63]. It needs to be investigated whether this protein plays a role singly or in combination with VAPA and VAPB in herpesvirus nuclear egress.

In future studies it will be interesting to also investigate the influence of a dominant negative version of VAPB. A proline to serine substitution (P56S) in VAPB, which was identified in an autosomal dominant form of amyotrophic lateral sclerosis, was shown to result in a nuclear envelope defect [41]. Blocking the normal function of VAPs by overexpressing the defective isoform might result in more drastic effects as single and double knockouts since several other cellular proteins might serve similar functions.

## Figures and Tables

**Table 1:** Primer and oligonucleotide sequences. Compatible 5′ overhangs for restriction enzyme BbsI used for cloning are underlined.

Name	Sequence (5′–3′)
VAPA_ctrl-seq_Fwd	TCGGGTTTAGATTTCTGCAGTT
VAPA_ctrl-seq_Rev	GCGTAATTTCATACACTGGCAA
VAPB_ctrl-seq_Fwd	ATCCTAACTGCTGCTAACTGGC
VAPB_ctrl-seq_Rev	CTCCAATTCTGAAATCCAGTCC
VAPA gRNA #1_Fwd	CACCAAACAGTCACAGTCGACCCT
VAPA gRNA #1_Rev	AAACAGGGTCGACTGTGACTGTTT
VAPA gRNA #2_Fwd	CACCGGCCTCACACAGTACCGGCG
VAPA gRNA #2_Rev	AAACCGCCGGTACTGTGTGAGGCC
VAPA gRNA #3_Fwd	CACCAACAGTGGAATTATTGACCC
VAPA gRNA #3_Rev	AAACGGGTCAATAATTCCACTGTT
VAPA gRNA #4_Fwd	CACCTCTTAAATTGCGAAATCCAT
VAPA gRNA #4_Rev	AAACATGGATTTCGCAATTTAAGA
VAPB gRNA #1_Fwd	CACCAACAGCGGGATCATTGACGC
VAPB gRNA #1_Rev	AAACGCGTCAATGATCCCGCTGTT
VAPB gRNA #2_Fwd	CACCGGGGCCTCCATTAACGTGTC
VAPB gRNA #2_Rev	AAACGACACGTTAATGGAGGCCCC
VAPB gRNA #3_Fwd	CACCGTGCTTTAAAGTGAAGACGA
VAPB gRNA #3_Rev	AAACTCGTCTTCACTTTAAAGCAC
HU6-F	ATAATTTCTTGGGTAGTTTGCAG

## Data Availability

The data presented in this study are available on request from the corresponding author.

## References

[1] Fields B.K.D. (2007). Fields Virology.

[2] Rothman J.E., Bursztyn-Pettegrew H., Fine R.E. (1980). Transport of the membrane glycoprotein of vesicular stomatitis virus to the cell surface in two stages by clathrin-coated vesicles. J. Cell Biol..

[3] Tran E.J., Wente S.R. (2006). Dynamic nuclear pore complexes: Life on the edge. Cell.

[4] McGeoch D.J., Rixon F.J., Davison A.J. (2006). Topics in herpesvirus genomics and evolution. Virus Res..

[5] Mettenleiter T.C. (2016). Breaching the Barrier-The Nuclear Envelope in Virus Infection. J. Mol. Biol.

[6] Wild P., Senn C., Manera C.L., Sutter E., Schraner E.M., Tobler K., Ackermann M., Ziegler U., Lucas M.S., Kaech A. (2009). Exploring the nuclear envelope of herpes simplex virus 1-infected cells by high-resolution microscopy. J. Virol..

[7] Leuzinger H., Ziegler U., Schraner E.M., Fraefel C., Glauser D.L., Heid I., Ackermann M., Mueller M., Wild P. (2005). Herpes simplex virus 1 envelopment follows two diverse pathways. J. Virol..

[8] Mettenleiter T.C. (2002). Herpesvirus assembly and egress. J. Virol..

[9] Skepper J.N., Whiteley A., Browne H., Minson A. (2001). Herpes simplex virus nucleocapsids mature to progeny virions by an envelopment --> deenvelopment --> reenvelopment pathway. J. Virol..

[10] Mettenleiter T.C., Muller F., Granzow H., Klupp B.G. (2013). The way out: What we know and do not know about herpesvirus nuclear egress. Cell. Microbiol..

[11] Johnson D.C., Baines J.D. (2011). Herpesviruses remodel host membranes for virus egress. Nat. Rev. Microbiol..

[12] Mettenleiter T.C., Klupp B.G., Granzow H. (2009). Herpesvirus assembly: An update. Virus Res..

[13] Hollinshead M., Johns H.L., Sayers C.L., Gonzalez-Lopez C., Smith G.L., Elliott G. (2012). Endocytic tubules regulated by Rab GTPases 5 and 11 are used for envelopment of herpes simplex virus. EMBO J..

[14] Klupp B.G., Granzow H., Mettenleiter T.C. (2000). Primary envelopment of pseudorabies virus at the nuclear membrane requires the UL34 gene product. J. Virol..

[15] Passvogel L., Klupp B.G., Granzow H., Fuchs W., Mettenleiter T.C. (2015). Functional characterization of nuclear trafficking signals in pseudorabies virus pUL31. J. Virol..

[16] Funk C., Ott M., Raschbichler V., Nagel C.H., Binz A., Sodeik B., Bauerfeind R., Bailer S.M. (2015). The Herpes Simplex Virus Protein pUL31 Escorts Nucleocapsids to Sites of Nuclear Egress, a Process Coordinated by Its N-Terminal Domain. PLoS Pathog..

[17] Schmeiser C., Borst E., Sticht H., Marschall M., Milbradt J. (2013). The cytomegalovirus egress proteins pUL50 and pUL53 are translocated to the nuclear envelope through two distinct modes of nuclear import. J. Gen. Virol..

[18] Bigalke J.M., Heldwein E.E. (2016). Nuclear Exodus: Herpesviruses Lead the Way. Annu. Rev. Virol..

[19] Desai P.J., Pryce E.N., Henson B.W., Luitweiler E.M., Cothran J. (2012). Reconstitution of the Kaposi’s sarcoma-associated herpesvirus nuclear egress complex and formation of nuclear membrane vesicles by coexpression of ORF67 and ORF69 gene products. J. Virol..

[20] Klupp B.G., Granzow H., Fuchs W., Keil G.M., Finke S., Mettenleiter T.C. (2007). Vesicle formation from the nuclear membrane is induced by coexpression of two conserved herpesvirus proteins. Proc. Natl. Acad. Sci. USA.

[21] Lorenz M., Vollmer B., Unsay J.D., Klupp B.G., Garcia-Saez A.J., Mettenleiter T.C., Antonin W. (2015). A single herpesvirus protein can mediate vesicle formation in the nuclear envelope. J. Biol. Chem..

[22] Bigalke J.M., Heuser T., Nicastro D., Heldwein E.E. (2014). Membrane deformation and scission by the HSV-1 nuclear egress complex. Nat. Commun..

[23] Sehl J., Portner S., Klupp B.G., Granzow H., Franzke K., Teifke J.P., Mettenleiter T.C. (2020). Roles of the different isoforms of the pseudorabies virus protein kinase pUS3 in nuclear egress. J. Virol..

[24] Mou F., Wills E., Baines J.D. (2009). Phosphorylation of the U(L)31 protein of herpes simplex virus 1 by the U(S)3-encoded kinase regulates localization of the nuclear envelopment complex and egress of nucleocapsids. J. Virol..

[25] Reynolds A.E., Wills E.G., Roller R.J., Ryckman B.J., Baines J.D. (2002). Ultrastructural localization of the herpes simplex virus type 1 UL31, UL34, and US3 proteins suggests specific roles in primary envelopment and egress of nucleocapsids. J. Virol..

[26] Klupp B.G., Granzow H., Mettenleiter T.C. (2001). Effect of the pseudorabies virus US3 protein on nuclear membrane localization of the UL34 protein and virus egress from the nucleus. J. Gen. Virol..

[27] Wagenaar F., Pol J.M., Peeters B., Gielkens A.L., de Wind N., Kimman T.G. (1995). The US3-encoded protein kinase from pseudorabies virus affects egress of virions from the nucleus. J. Gen. Virol..

[28] Rothballer A., Schwartz T.U., Kutay U. (2013). LINCing complex functions at the nuclear envelope: What the molecular architecture of the LINC complex can reveal about its function. Nucleus.

[29] Klupp B.G., Hellberg T., Granzow H., Franzke K., Dominguez Gonzalez B., Goodchild R.E., Mettenleiter T.C. (2017). Integrity of the Linker of Nucleoskeleton and Cytoskeleton Is Required for Efficient Herpesvirus Nuclear Egress. J. Virol..

[30] Holper J.E., Klupp B.G., Luxton G.W.G., Franzke K., Mettenleiter T.C. (2020). Function of Torsin AAA+ ATPases in Pseudorabies Virus Nuclear Egress. Cells.

[31] Atai N.A., Ryan S.D., Kothary R., Breakefield X.O., Nery F.C. (2012). Untethering the nuclear envelope and cytoskeleton: Biologically distinct dystonias arising from a common cellular dysfunction. Int. J. Cell Biol..

[32] Nery F.C., Zeng J., Niland B.P., Hewett J., Farley J., Irimia D., Li Y., Wiche G., Sonnenberg A., Breakefield X.O. (2008). TorsinA binds the KASH domain of nesprins and participates in linkage between nuclear envelope and cytoskeleton. J. Cell Sci..

[33] Hirohata Y., Arii J., Liu Z., Shindo K., Oyama M., Kozuka-Hata H., Sagara H., Kato A., Kawaguchi Y. (2015). Herpes Simplex Virus 1 Recruits CD98 Heavy Chain and beta1 Integrin to the Nuclear Membrane for Viral De-Envelopment. J. Virol..

[34] Farnsworth A., Wisner T.W., Webb M., Roller R., Cohen G., Eisenberg R., Johnson D.C. (2007). Herpes simplex virus glycoproteins gB and gH function in fusion between the virion envelope and the outer nuclear membrane. Proc. Natl. Acad. Sci. USA.

[35] Vallbracht M., Backovic M., Klupp B.G., Rey F.A., Mettenleiter T.C. (2019). Common characteristics and unique features: A comparison of the fusion machinery of the alphaherpesviruses Pseudorabies virus and Herpes simplex virus. Adv. Virus Res..

[36] Klupp B., Altenschmidt J., Granzow H., Fuchs W., Mettenleiter T.C. (2008). Glycoproteins required for entry are not necessary for egress of pseudorabies virus. J. Virol..

[37] Lee C.P., Liu P.T., Kung H.N., Su M.T., Chua H.H., Chang Y.H., Chang C.W., Tsai C.H., Liu F.T., Chen M.R. (2012). The ESCRT machinery is recruited by the viral BFRF1 protein to the nucleus-associated membrane for the maturation of Epstein-Barr Virus. PLoS Pathog..

[38] Arii J., Watanabe M., Maeda F., Tokai-Nishizumi N., Chihara T., Miura M., Maruzuru Y., Koyanagi N., Kato A., Kawaguchi Y. (2018). ESCRT-III mediates budding across the inner nuclear membrane and regulates its integrity. Nat. Commun..

[39] Pawliczek T., Crump C.M. (2009). Herpes simplex virus type 1 production requires a functional ESCRT-III complex but is independent of TSG101 and ALIX expression. J. Virol..

[40] Saiz-Ros N., Czapiewski R., Epifano I., Stevenson A., Swanson S.K., Dixon C.R., Zamora D.B., McElwee M., Vijayakrishnan S., Richardson C.A. (2019). Host Vesicle Fusion Protein VAPB Contributes to the Nuclear Egress Stage of Herpes Simplex Virus Type-1 (HSV-1) Replication. Cells.

[41] Tran D., Chalhoub A., Schooley A., Zhang W., Ngsee J.K. (2012). A mutation in VAPB that causes amyotrophic lateral sclerosis also causes a nuclear envelope defect. J. Cell Sci..

[42] Lev S., Ben Halevy D., Peretti D., Dahan N. (2008). The VAP protein family: From cellular functions to motor neuron disease. Trends Cell Biol..

[43] Nishimura Y., Hayashi M., Inada H., Tanaka T. (1999). Molecular cloning and characterization of mammalian homologues of vesicle-associated membrane protein-associated (VAMP-associated) proteins. Biochem. Biophys. Res. Commun..

[44] Teuling E., Ahmed S., Haasdijk E., Demmers J., Steinmetz M.O., Akhmanova A., Jaarsma D., Hoogenraad C.C. (2007). Motor neuron disease-associated mutant vesicle-associated membrane protein-associated protein (VAP) B recruits wild-type VAPs into endoplasmic reticulum-derived tubular aggregates. J. Neurosci..

[45] McCune B.T., Tang W., Lu J., Eaglesham J.B., Thorne L., Mayer A.E., Condiff E., Nice T.J., Goodfellow I., Krezel A.M. (2017). Noroviruses Co-opt the Function of Host Proteins VAPA and VAPB for Replication via a Phenylalanine-Phenylalanine-Acidic-Tract-Motif Mimic in Nonstructural Viral Protein NS1/2. mBio.

[46] Hamamoto I., Nishimura Y., Okamoto T., Aizaki H., Liu M., Mori Y., Abe T., Suzuki T., Lai M.M., Miyamura T. (2005). Human VAP-B is involved in hepatitis C virus replication through interaction with NS5A and NS5B. J. Virol..

[47] Arii J. (2021). Host and Viral Factors Involved in Nuclear Egress of Herpes Simplex Virus 1. Viruses.

[48] Kopp M., Klupp B.G., Granzow H., Fuchs W., Mettenleiter T.C. (2002). Identification and characterization of the pseudorabies virus tegument proteins UL46 and UL47: Role for UL47 in virion morphogenesis in the cytoplasm. J. Virol..

[49] Mettenleiter T.C. (2006). Intriguing interplay between viral proteins during herpesvirus assembly or: The herpesvirus assembly puzzle. Vet. Microbiol..

[50] Kaplan A.S., Vatter A.E. (1959). A comparison of herpes simplex and pseudorabies viruses. Virology.

[51] Yates A.D., Achuthan P., Akanni W., Allen J., Allen J., Alvarez-Jarreta J., Amode M.R., Armean I.M., Azov A.G., Bennett R. (2020). Ensembl 2020. Nucleic Acids Res..

[52] Labun K., Montague T.G., Krause M., Torres Cleuren Y.N., Tjeldnes H., Valen E. (2019). CHOPCHOP v3: Expanding the CRISPR web toolbox beyond genome editing. Nucleic Acids Res..

[53] Hubner A., Petersen B., Keil G.M., Niemann H., Mettenleiter T.C., Fuchs W. (2018). Efficient inhibition of African swine fever virus replication by CRISPR/Cas9 targeting of the viral p30 gene (CP204L). Sci. Rep..

[54] Graham F.L., van der Eb A.J. (1973). A new technique for the assay of infectivity of human adenovirus 5 DNA. Virology.

[55] Dong R., Saheki Y., Swarup S., Lucast L., Harper J.W., De Camilli P. (2016). Endosome-ER Contacts Control Actin Nucleation and Retromer Function through VAP-Dependent Regulation of PI4P. Cell.

[56] Schindelin J., Arganda-Carreras I., Frise E., Kaynig V., Longair M., Pietzsch T., Preibisch S., Rueden C., Saalfeld S., Schmid B. (2012). Fiji: An open-source platform for biological-image analysis. Nat. Methods.

[57] Mettenleiter T.C. (1989). Glycoprotein gIII deletion mutants of pseudorabies virus are impaired in virus entry. Virology.

[58] Hagen C., Dent K.C., Zeev-Ben-Mordehai T., Grange M., Bosse J.B., Whittle C., Klupp B.G., Siebert C.A., Vasishtan D., Bauerlein F.J. (2015). Structural Basis of Vesicle Formation at the Inner Nuclear Membrane. Cell.

[59] Walzer S.A., Egerer-Sieber C., Sticht H., Sevvana M., Hohl K., Milbradt J., Muller Y.A., Marschall M. (2015). Crystal Structure of the Human Cytomegalovirus pUL50-pUL53 Core Nuclear Egress Complex Provides Insight into a Unique Assembly Scaffold for Virus-Host Protein Interactions. J. Biol. Chem..

[60] Lye M.F., Sharma M., El Omari K., Filman D.J., Schuermann J.P., Hogle J.M., Coen D.M. (2015). Unexpected features and mechanism of heterodimer formation of a herpesvirus nuclear egress complex. EMBO J..

[61] Zeev-Ben-Mordehai T., Weberruss M., Lorenz M., Cheleski J., Hellberg T., Whittle C., El Omari K., Vasishtan D., Dent K.C., Harlos K. (2015). Crystal Structure of the Herpesvirus Nuclear Egress Complex Provides Insights into Inner Nuclear Membrane Remodeling. Cell Rep..

[62] Bigalke J.M., Heldwein E.E. (2015). Structural basis of membrane budding by the nuclear egress complex of herpesviruses. EMBO J..

[63] Di Mattia T., Wilhelm L.P., Ikhlef S., Wendling C., Spehner D., Nomine Y., Giordano F., Mathelin C., Drin G., Tomasetto C. (2018). Identification of MOSPD2, a novel scaffold for endoplasmic reticulum membrane contact sites. EMBO Rep..

[64] Eisenberg-Bord M., Shai N., Schuldiner M., Bohnert M. (2016). A Tether Is a Tether Is a Tether: Tethering at Membrane Contact Sites. Dev. Cell.

